# A Case of Recurrent Respiratory Papillomatosis With Lung Involvement and Malignant Transformation

**DOI:** 10.7759/cureus.24370

**Published:** 2022-04-22

**Authors:** Alejandra Valdivia Padilla, Eduardo Tellez-Garcia, Horiana Grosu

**Affiliations:** 1 Internal Medicine, Instituto Tecnologico y de Estudios Superiores de Monterrey, Monterrey, MEX; 2 Pulmonary Medicine, MD Anderson Cancer Center, Houston, USA

**Keywords:** human papillomavirus (hpv), tracheoesophageal fistula, bronchoscopy, papillomas, recurrent respiratory papillomatosis

## Abstract

Recurrent respiratory papillomatosis is a rare and complex progression of the disease due to the human papillomavirus (HPV). In this case report, we present the findings of a 53-year-old male who was diagnosed with obstructing respiratory papillomatosis of the trachea and underwent several procedures until the disease progressed to squamous cell carcinoma. Our objective with this case report is to contribute to a broader understanding of this disease by reporting a clinical case.

## Introduction

Recurrent respiratory papillomatosis (RRP) is a challenging disease to treat. It is the result of infection by human papillomavirus (HPV) [1,2], most commonly involving the subtypes HPV-6 and HPV-11 [3-5]. Although any part of the aerodigestive tract can be affected, the most common location of papillomas is the larynx [1,2]. The clinical course varies greatly, with some cases resolving spontaneously and others recurring multiple times and requiring several surgical procedures [2,6]. The most common symptoms are hoarseness, dyspnea, cough, and dysphagia, among others [7]. Papillomas are usually benign, but dysplasia and malignant transformation sometimes can be seen [2,8]. Pulmonary lesions tend to be unusual and represent less than 1% of all lung neoplasms [2,9]; however, when the progression of the disease becomes severe, these lesions could cause damage to the pulmonary parenchyma [2,9].

## Case presentation

**Figure 1:**
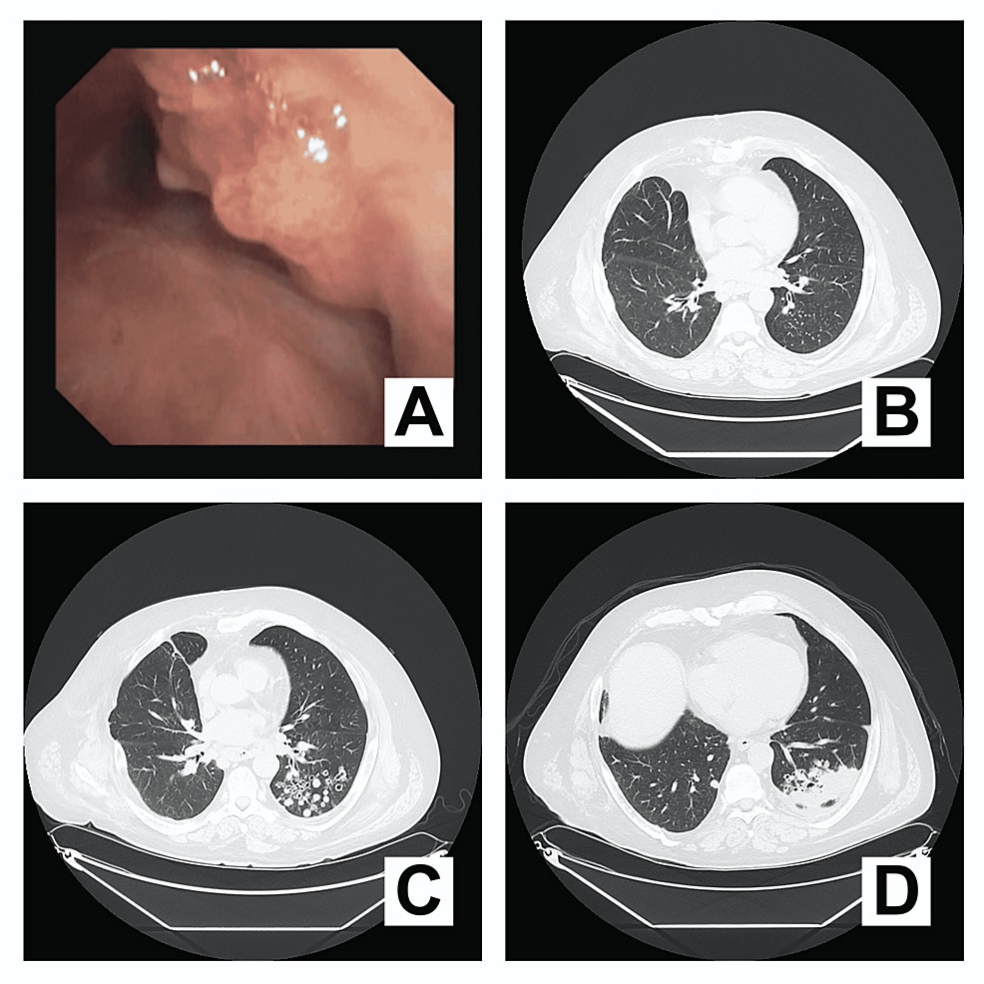
Papillomas in the trachea (A). CT scans showing bilateral small lung nodules (B), progression to cavitary nodules (C), and progression to squamous cell carcinoma (confirmed by biopsy) (D).

A 53-year-old male was diagnosed with obstructing RRP of the trachea in 2018 and underwent repeated bronchoscopic debulking procedures. These were complicated by a tracheoesophageal fistula, which required several surgical interventions. Thereafter, the papillomas of the trachea recurred, and the patient had repeated bronchoscopic removals (Figure 1A). A computed tomography (CT) showed bilateral small lung nodules (Figure 1B). On subsequent CT, nodules progressed to cavitary nodules (Figure 1C). Biopsy of the cavitary nodules showed focal atypical squamous proliferation. Given the progression of the cavitary nodules (Figure 1D), a repeat biopsy was performed and squamous cell carcinoma was reported. In addition to surgery, the patient started therapy with carboplatin and pembrolizumab. Overall, the patient had a total of 14 therapeutic bronchoscopies before developing pulmonary disease that progressed to squamous cell carcinoma. Unfortunately, the patient’s cancer progressed further, and he was considered for hospice.

## Discussion

Though there is no cure for RRP [1,2], surgery remains the mainstay therapy [1,8], while the recurrence rate remains a challenge [2,10]. According to literature after four surgical procedures in approximately 12 months [1,2,8], there is a need to establish adjunctive therapies [8]. In cases where the patency of the airway is severely compromised, tracheotomy is often necessary [2,8]. HPV vaccination plays a key role in RRP prevention [1,11,12], as studies have shown a decrease in incidence and prevalence, it also has demonstrated a potential therapeutic use for the vaccine [11-13]. Immunotherapy also has a potential role in managing RRP, owing to the pathophysiology of RRP and the new evidence that HPV infection causes an immune dysregulation, representing a promising line of research [1,11,12].

## Conclusions

Clinicians should be aware of the possible complications of HPV in order to establish complementary therapies in patients susceptible to developing RRP. Furthermore, the vaccine continues to be the main preventive measure to avoid this disease. However, as with immunotherapy, more research is needed to determine the extent to which it can be used as a therapeutic measure.
